# Exploring Politics and Contestation in the Policy Process: The Case of Zambia’s Contested Community Health Strategy

**DOI:** 10.34172/ijhpm.2021.145

**Published:** 2021-10-23

**Authors:** Joseph M. Zulu, Maligzani P. Chavula, Adam Silumbwe, Margarate N. Munakampe, Chama Mulubwa, Wanga Zulu, Charles Michelo, Helen Schneider, Uta Lehmann

**Affiliations:** ^1^Department of Health Promotion and Education, School of Public Health, The University of Zambia, Lusaka, Zambia.; ^2^Department of Health Policy and Management, School of Public Health, The University of Zambia, Lusaka, Zambia.; ^3^The Ministry of Health, Lusaka, Zambia.; ^4^Department of Epidemiology and Biostatistics, School of Public Health, The University of Zambia, Lusaka, Zambia.; ^5^School of Public Health, University of the Western Cape, Cape Town, South Africa.; ^6^South African Medical Research Council Health Services to Systems Unit, University of the Western Cape, Cape Town, South Africa.

**Keywords:** Community Health Strategy, Community Health System, Politics, Policy Development

## Abstract

There have been increased calls for low- and middle-income countries to develop community health systems (CHS) policies or strategies. However, emerging global guidance brackets the inherent complexity and contestation of policy development at the country level. This is explored through the case of Zambia’s 5-year Community Health Strategy (CH Strategy), formulated in 2017 and then summarily withdrawn and reissued two years later, with largely similar content. This paper examines the events, actors, and contexts behind this abrupt change in the Strategy, through an analysis of documentary sources and interviews with 21 stakeholders involved in the policy process. We describe an environment of contestation, characterised by numerous international partners weighing in on the CH Strategy, interfacing with shifting loci of responsibility for the CHS in the Ministry of Health (MoH). Despite the rhetoric of participation, providers and communities played no part in the policy process. These dynamics created the conditions for the abrupt change in strategy, illustrating the inherently fraught and political nature of policy development on the CHS in many countries. Going forward, we conclude that paying attention to processes of CHS policy development, and in particular the interaction between events, actors, and contexts, is as important as ensuring meaningful policy content.

## Background

Community health systems (CHS) are the subject of growing interest based on their potential to leverage different community resources, enhance primary healthcare and advance population well-being in attaining universal health coverage.^1^ The literature suggests that CHS can extend preventive and curative health services into communities through integrated community-level approaches,^2^ while also widening participation, collective action, and accountability.^3^ Low- and middle-income countries that have invested in CHS have shown gains in health status.^4^ Similarly, multifaceted health needs in high-income countries have shifted thinking away from hospicentric and curative approaches towards more flexible and person-centred models of primary care.^5,6^

Despite the growing momentum in support of CHS, community health (CH) programs experience many challenges. These include underfunding and the difficulty of bridging the gap between idealized policy and implementation realities,^3^ a wide array of community programs involving multiple stakeholders, extensive fragmentation and complex community contexts.^3,7^ Fragmentation is partly due to the way programs and initiatives are funded – as vertical and disease-specific, and partly to the lack of coordination mechanisms.^7^ Further, the understanding of the CHS is quite varied within and across countries and in the health systems research fraternity.^8^ This understanding ranges from the narrow view of CHS as heavily focused on local community volunteer programs, to broader concepts that encompass all of society’s efforts aimed at improving population wellbeing.^2,8^

With the growing global interest in the role of CHS, donors and international agencies are engaging Ministries of Health in multiple countries (including Zambia) to develop CH policy. An example of this is the “*Community Health Roadmap [that] aims to elevate national community health priorities and create a common agenda for investments in community health to strengthen primary healthcare” *(https://www.communityhealthroadmap.org/). While there have been increased calls for national governments to develop strategies, most of the guidance has concerned the content of policy and little regarding CH strategy development, drivers and stakeholder participation.^9^

 Amid increasing donor support and calls for strengthening CHS, Zambia developed and launched a 5-year CH Strategy in 2017, expected to run until 2021. However, two years later, in 2019, this Strategy was summarily withdrawn and replaced with a new 3-year Strategy (2019 to 2021) covering very similar content as the first one.

This paper explores these developments – the actors and processes involved and what they reveal about CHS policy-making as an arena of actor interests and contestation at a country level. Empirical literature shows that developing coherent CH policies is shaped by different levels of power and agendas for the CHS, derived from political authority, financial resources and technical expertise.^10^ Actors’ power or position in the political and administrative hierarchy may play a disproportionate role in shaping the policy process and content,^11^ while other key actors are silenced in the process.^12^ Furthermore, a proliferation of internal and external actors all pursuing their agendas contributes to the complexity and contestation of policy processes.^10^

 We analyse the case of Zambia to illustrate the political nature of the CHS policy process and specifically the increasingly crowded and contested stakeholder environment involved in the policy process. This paper aimed to explore the events, actors, and contexts behind the abrupt change in the CH Strategy. We begin by outlining the context driving the need to develop the Zambian Strategy, describe the methodology used to collect data for the case study, then report on the study findings, and discuss their implications for future CH policy development.

###  Context Driving the Need to Develop the Strategy 

 Zambia is a lower-middle-income country with a population of about 17 million people. About 60% of Zambians live in rural areas in extreme poverty. Health services are provided by the public health sector with government-owned and run facilities, faith-based not-for-profit providers, mine health facilities, private-for-profit providers, community-based organizations and traditional practitioners. Recent figures showed that 46% of rural households in Zambia lived outside a radius of 5 km from a health facility, compared to only 1% of urban households. The public sector health delivery is structured as a three-tier pyramidal referral system. This system consists of primary healthcare (health posts, health centres and district hospitals), secondary healthcare (provincial referral hospitals), and tertiary healthcare (teaching and specialised hospitals) (2019-2021 National Community Health Strategy).

 To address disparities and geographic challenges concerning access to healthcare, Zambia launched the decentralization of health services management to the district level in the 1990s. These decentralization reforms emphasized the adoption of community involvement in health as a key strategy, based on the Alma-Alta Declaration on Primary Healthcare of 1978. Through the National Health Services Act of 1996, the government established community representative structures at all levels of healthcare and delegated significant decision-making powers to the District Health Management Teams, Health Centre Committees and Health Post Committees. The Ministry of Health (MoH) collaborates with other Ministries and non-state actors (including faith-based providers) in delivering services at national, provincial, district and community levels.

In practice, CHS in Zambia are highly fragmented (2019 Community Health Strategy) involving community-based volunteers (CBVs), an array of donor-funded vertical projects and disease-specific programs, and a formalised cadre, the community health assistant (CHA). The CHAs were introduced by the MoH in 2010 through its National Community Health Worker Strategy to reposition and expand the available cadre of frontline workers in CHS.^11^ The CHAs, unlike CBVs, are on the government payroll and receive a standardized one-year training. These developments brought some improvement with regard to community interventions and programming. However, for the most part the CHS remains poorly regulated and fragmentation problems persist, impacting joint planning, implementation and mutual accountability.

 The CH Strategy was formulated in 2017 partly in response to these problems, to guide the strengthening of coordination mechanisms and to expand the provision of preventive, promotive and minor curative services at the community level. This was followed by the establishment of the CH Unit in 2018, with the mandate to improve national coordination of CH initiatives and enhance CH promotion. The Directorate of Health Promotion, Environment and Social Determinants led and oversaw the process of establishing the Unit, drawing on experiences in Ghana and other countries, and informed by the recommendations of the National Health Strategy Plan 2017-2021 and the Zambia Vision 2030. The locus of responsibility for the CH Unit was subsequently shifted when senior management in the MoH decided to move the Unit to the Directorate of Public Health and Research, based on the understanding that CH was broader than health promotion. The Assistant Director of the newly located CH Unit then coordinated the development of the revised 2019 Strategy. These various changes introduced new sets of governmental actors and interests in the CHS.

## Methods

###  Study Design 

We used a case study approach to explore the complexity of developing CH strategies and policies, with the case being of actors and processes in CHS policy-making. According to Yin case studies are best suited for exploring the ‘why’ and ‘how’ questions in health policy change.^13^ This approach was considered as appropriate for this study because the CH Strategy was developed within a complex context, which involved social interactions among multiple stakeholders with different agendas. This approach enabled us to understand how and why the contextual realities shaped the process of developing the CH Strategy.

###  Study Data Collection 

 Data collection took place in March 2020. First, we engaged the MoH to obtain general information (including meeting minutes) relating to the development of both the 2017 and 2019 National Community Health Strategies. Using the meeting minutes, we purposively selected 21 participants from various government departments under the MoH and other stakeholders who had been involved in developing the two strategies. Twelve of the respondents were female while the rest were male. The participants who were selected were those who were in Zambia and available to be interviewed. All those who sampled agreed to participate in the interviews. Six of the participants were from the MoH, 5 funding partners, and 20 implementing partners. Seven people who had participated in the 2017 policy development were interviewed, while the remainder were part of the 2019 process. Five respondents had participated in both processes.

 All the interviews were conducted in English by experienced qualitative data collectors. The interviews lasted between 35 and 60 minutes. Data from the interviews were triangulated by reviewing meeting minutes, the 2017 and 2019 CH Strategy documents and a presentation to senior management on the need to withdraw the first CH Strategy.

###  Data Analysis

All audio recordings were transcribed verbatim and imported into NVivo version 12 for coding and analysis. Thematic analysis, as described by Braun and Clarke was done using a coding-framework.^14^ Initial coding was done by four co-authors (AS, MPC, CM, MM) separately to ensure inter-coder reliability.^15^ Initial codes were then extensively discussed with all the co-authors and later merged into sub-themes. The sub-themes were then refined to develop themes. Content analysis of the 2017 and 2019 CH Strategies was done by JMZ. The analysis involved systematically mapping the similarities, differences and gaps in the two policies (Table).

**Table:** Similarities and Differences Between the 2017 and 2019 Strategies

**Similar Thematic Areas in the 2017 and 2019 Strategies **	**Additional (New) Thematic Areas in the 2019 Strategy Only **	**Gaps in 2019 Strategy **
Strengthening of governance of CHS through enhanced involvement of local government councils, participation of headmen in NHC meetings	Inclusion of a specific strategy for increasing access to services including demand creation, development of the CH service package	Limited focus on other community leaders such as leaders of clubs, religious and traditional leaders
Implementation of devolution guidelines including adoption of “bottom-up” decision-making, development of a clear legal framework for NHCs and health centre committees, and formalizing the role of CBVs	Increasing the annual CH budget for Zambia by 100% per year between 2019 and 2021 through strengthening the capacity of the CH Unit	No clear roles for other key Ministries such the Ministry of Community Development and Social Welfare, Agriculture, Chiefs and Traditional Affairs
Development of regulations and guidelines for CHS	Developing innovations in CH	
Strengthening CHS organizational structures through development of legal and regulatory framework for CH structures		
Building a motivated, skilled, equitably distributed CH workforce		
Strengthening CH decision-making by ensuring timely availability of data		

Abbreviatiopns: CH, community health; CHS, community health systems; CBVs, community-based volunteers; NHC, neighborhood health committee.

The trustworthiness of findings was enhanced through attending to aspects of credibility, dependability and transferability of the findings.^16^ To enhance the credibility and dependability of findings, we comprehensively reviewed the data and coded it as a team. Four co-authors independently reviewed the codes and categories and then discussed their insights to develop the final themes. We also shared or validated the preliminary results with two members that were involved in developing the CH Strategy. Transferability was enhanced by reviewing the content of the CH Strategies, providing a description of context, process and content of the 2017 and 2019 CH Strategies and providing quotations from the different study participants.

 A challenge of the study was that most of the people that had participated in the first strategy were not available for interview as they changed locations (and somewhat reflective of an unstable policy environment). Further, some respondents requested that some information such as positions/organizations of the actors who triggered the revision process of the CH Strategy, not be included as they considered it too sensitive.

## Results

 This section begins with an outline of the mandated procedures for policy development, followed by how the CH Strategy was developed. We then discuss the repeated attempts by the MoH to grapple with multiple actors and processes in CHS policy-making.

###  Mandated Procedures 

 In Zambia, the mandated approach to policy development starts with the generation of evidence through a review of existing literature and the consultation of key stakeholders at the national, provincial, district and community levels. The consultation process is preceded by a mapping of the key stakeholders, led by the host Ministry and a Policy Coordination Committee. The multisectoral consultation, in the context of the CH Strategy, involves state and non-state actors who collaborate with the MoH in delivering services at national, provincial, district and community levels. Thereafter, a policy brief is developed and validated by various stakeholders. Once validated, the policy is drafted and circulated to the relevant state and non-state actors for review. Feedback from the Inter-Ministerial consultation is submitted to the Ministry that will host the policy, for final validation. The Policy Coordination Committee thereafter conducts quality assurance before the policy is submitted to the Cabinet for approval. Once approved, the host Minister signs off the policy followed by the Permanent Secretary.

###  Developing and Revising the Community Health Strategy 

As indicated earlier, Zambia formulated the its first CH Strategy in 2017. This was steered by the Health Promotion Unit in the MoH with the assistance of a European Union-funded consultant. The MoH, local and international non-governmental organizations, and some of the funding agencies and UN bodies participated in the development of the first CH Strategy (Figure). The Strategy was supposed to run from 2017 to 2021. Of note is that the CH Strategy process did not follow all the mandated steps in the policy process outlined above, specifically in terms of stakeholder consultation and signing off procedures, thus rendering it vulnerable.

**Figure:**
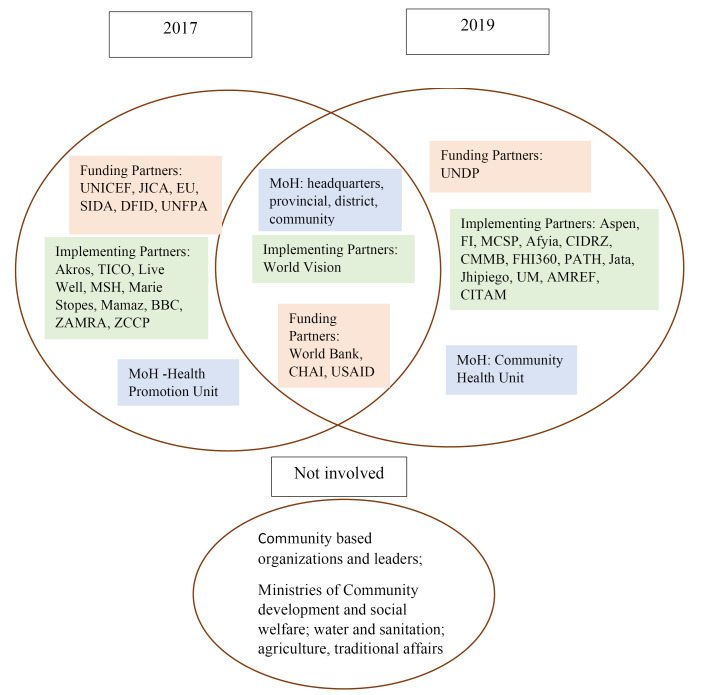
Stakeholders Involved in Developing the 2017 and 2019 Community Health Strategies. Abbreviations: UNICEF, United Nations Children’s Fund; EU, European Union; UNFPA, United Nations Population Fund; MoH, Ministry of Health; USAID, United States Agency for International Development; UNDP, United Nations Development Programme; JICA, Japan International Cooperation Agency; DFID, Department for International Development; SIDA, Swedish International Development Cooperation Agency; TICO, Tokushima International Cooperation; MSH, management sciences for health; ZAMRA, Zambia Medicines Regulatory Authority; ZCCP, Zambia Centre for Communication Programmes; CHAI, Clinton Health Access Initiative; MCSP, Maternal and Child Survival Program; CIDRZ, Centre for Infectious Disease Research in Zambia; CMMB, Catholic Medical Mission Board; FHI360, Family Health International; PATH, Program for Appropriate Technology in Health; Jhipiego, Johns Hopkins Program for International Education in Gynecology and Obstetrics; UM, University of Maryland; AMREF, African Medical and Research Foundation; CITAM, Community Initiative For TB, HIV/AIDS & Malaria.

###  Declaring the First Strategy “Null and Void”

After launching the first CH Strategy, some funding agencies complained to the Minister of Health and senior management at the MoH that they ‘did not feel consulted,’ andexpressed discontent that the Strategy did not adequately take into account their views *(Presentation to Senior Management, July 2019)*. The funding agencies made some specific critiques: that the activities in the strategy were not sufficiently detailed, thereby making it difficult for the MoH to develop an implementation budget; that the activities and targets in 2017 strategy were too ambitious; and that the strategy was not aligned to the National Health Strategic Plan (2017-2021).

*“She (the EU-funded consultant) did a good job but objectives of the strategy were not in line with National Health Strategy – and also some views from key stakeholders were not included” *[Key informant interview (KII) 20, 2019 strategy development participant].

 Overall, the first strategy was portrayed as too complicated and impractical to implement at the community level. In addition, the process of approving and launching the strategy reportedly did not did not include key ‘senior political actors’ in signing-off the policy, as prescribed by the policy development process.

*“Largely, the decision to revise the strategy was political because some senior political actors did not look at the final document before it was signed”* [KII 19, 2019 strategy development participant].

 The complaints regarding the gaps in the first CH strategy that were highlighted by funders, coupled with the lack of involvement of senior political actors in approving and signing-off the document led to the declaration of the Strategy as ‘null and void.’ However, this language was contested by some, ultimately resulting in a compromise - to revise the first strategy and not discard it completely. While senior political actors and some donors advocated for the development of a new CH Strategy, other donors and local implementing partners, MoH officials, including those from the Health Promotion Department argued for the first CH Strategy to be maintained. Some donors reportedly warned that they would not fund the process of developing another strategy as they felt that the first strategy contained more ‘relevant issues’ which were important to CH.

*“At first, it was declared null and void, but others said that we cannot declare it null and void because it has some relevant issues. They suggested that it is better to look at the earlier version and refine it further. Some funders said that if the term null and void is, then they would not fund the new process” *[KII 21, 2017 and 2019 strategy development participant].

A review of the content of the two strategies shows indeed that they are not substantively different (Table). The second policy adopted most of the content that was outlined in the first strategy, concerning the governance of CHS, devolution, as well as regulations and guidelines for CH. Also, both strategies referenced similar policy guidelines namely the National Health Policy 2013; Zambia Vision 2030; The National Health Policy 2012; The Seventh National Development Plan; The National Health Strategic Plan (2017-2021); and The Community Health Worker Strategy (2010).

Although the 2019 strategy was perceived by some respondents as more comprehensive than the 2017 strategy, neither addressed issues related to the operations of the local context, actors and multisectoral collaboration. For example, there is limited focus on how the other community leaders such as leaders of clubs and the religious and traditional leaders would be involved in health service delivery (Table). Also, neither policy development processes included community or CHA representatives or actors from other government sectors.

###  Politics of Stakeholder Engagement in Developing the Community Health Strategy 

The CH policy process in Zambia was characterized by contestation amidst a proliferation of stakeholders (Figure).

 As shown in Figure, altogether, 40 actors were involved in the CH Strategy development processes: six stakeholders from the MoH, 10 funding partners, and 24 implementing partners. Funding partners included the United Nations Children’s Fund, Japan International Cooperation Agency, European Union, Swedish International Development Cooperation Agency, Department for International Development, the United Nations Population Fund, World Bank, Clinton Health Access Initiative, the United States Agency for International Development and the United Nations Development Programme. Implementing partners were international and local non-governmental organizations, of which 10 participated in developing the first CH Strategy, 13 participated in the second process, and one in both. Overall, only 8 stakeholders participated in both processes.

 Several reasons were provided as to why some of the original stakeholders did not attend meetings for the development of the 2019 CH Strategy, despite being invited. The Health Promotion Unit and some stakeholders from the MoH and other sectors shunned the revision process as they did not agree with the decision made by senior ministry officials to shift the location of the new CH Unit to a new division. They believed that the newly-located CH Unit had ‘grabbed’ the mandate of coordinating the process from the Health Promotion Unit.

*“People have not fully understood that the mandate of the Community Health Unit. As such others felt the Unit had grabbed the strategy from them” *[KII 19, 2019 strategy development participant].

 Other stakeholders did not participate because they felt that the Community Heath Unit was too new and did not have the technical capacity to manage the task of revising the CH Strategy.

 The implementing partners and donors did not attend the meetings because they felt that the first strategy had not been given sufficient time to be implemented. This short period denied the stakeholders an opportunity to draw lessons from the first Strategy to inform further development processes of CH strategies.

*“Honestly, if you look at the Community Health Strategy, we do not really have time. You know after a document like that has been done, you need time to implement the activities that you have been spelt out*” [KII8, 2017 and 2019 strategy development participant].

 Disagreement on the CH Strategy content also led some people to shun the revision process. Those who were happy with the content of the first strategy opted out of participation in the revision process.

*“Some stakeholders thought the first concept was good enough, so they opted not to participate in the revision process”* [KII5, 2017 and 2019 strategy development participant].

## Discussion

The Zambian experience of developing a CH strategy has shown that the process of coordinating and aligning actors and policies for the CHS is complex. This complexity manifested through the multiple partners contesting the CH Strategy development process, which resulted into adoption and rejection of policy within a space of two years of developing the first policy. This complexity was compounded by changes and contestation in the MoH itself, which struggled to steer this diverse stakeholder terrain. The development of a new CH Unit at the MoH, and transferring of the mandate to coordinate the process of revising the strategy from the Health Promotion Unit to this new Unit also contributed to complex inter-unit contestation, creating a proliferation of governmental and non-governmental actors pursuing their own interests. What emerges from such contestation, such as adoption and rejection of policy, is ultimately a function of the degree of power people bring to the policymaking process, related to political authority, donor (financial resources) and bureaucratic/technical know how.^10^ In such power struggles, actors key to implementation at lower levels of the system may become marginalized, as happened in Zambia.

These experiences suggest the need to recognize the inherently political nature of CH policy development, and for explicit attention to ensuring coherent processes of policy development. CHS policy processes need to adequately map and consider all the stakeholders and their roles in CH at the onset of policy development using comprehensive strategies. An inclusive and flexible approach to mapping actors is important as the boundaries and implementation pathways of community programs can be more varied and porous than initially anticipated.^6^ In the context of Zambia, this would mean including stakeholders beyond the MoH as well as other government sectors such as housing, education, and social development.^2,17,18^ Better engagement processes could help also address additional key challenges in terms of policy and practice for the CHS, such as unclear community workforce planning, identity and link to the health system.^3^

What has been presented in this paper speaks to the complexities, politics and contestation at the central level of policy development. We note that better policymaking and stakeholder involvement also has to take into account the interests of implementation actors and local contexts. Understanding of contextual implementation realities in the policy process is key as communities are sites of transformation whose expertise, capacities and ownership, coupled with external support shape the course and pattern of health innovations and outcomes.^3^ If not fully explored and mapped, these realities could affect scaling up of CH efforts, including policies as CHS policies are complex – they *involve a large number of diverse elements, that interact dynamically, often in non-linear ways, informed by direct and indirect feedback, in open systems with memory and adaptive capacities.*^3^ It is therefore important to look ‘into CHS’ as the site of formal programming as well as shifting the emphasis from the what (design) to the how (implementation) of programs. It further important to put in place systems that take the perspectives, priorities and actions of communities (rather than the health system) as starting points in designing CH programs. This entails taking into account how diverse actors interpret, respond and adapt to changes that are triggered by community interventions and programs.^3^

 One main limitation of this study was the lack of voices from various stakeholders that were excluded from the CH Strategy development process. We recommend that future studies include pay particular attention to such stakeholders in order to understand what and how they might have contributed to shaping the CH Strategy.

## Conclusion

 Within a space of two years, Zambia developed two CH Strategies. The paper has documented the highly complex development process of the CH Strategy in Zambia. The complexity and contestation of the policy process were created by a proliferation of internal and external actors all pursuing their own agendas. Stakeholders struggled to agree on the right content and process of developing the Strategy. Such disagreement resulted in many stakeholders who participated in developing the first Strategy shunning the revision process. The politics surrounding the strategy development process may have negative implications for reducing fragmentation in CHS. Thus, as countries develop their strategies, it is important from the onset to systematically map and involve all actors in CHS including roles, interests and power if such strategies are to be responsive to CHS.

## Acknowledgements

 This work received financial support from the Swedish Research Council Research Link project grant number 2016–05830. We are grateful to the MoH for allowing us to undertake this study, and also to all the respondents who participated in this study.

## Ethical issues

 Ethical approval to conduct the study was sought from the Excellence in Research Ethics and Science Converge (ERES) ethics committee. Permission to conduct the study was granted by the MoH on March 3, 2020. Informed consent was sought from all the participants before the interviews were conducted. Participation was voluntary, non-remunerable and consent to be recorded was sought separately from consent to take part in the study.

## Competing interests

 Authors declare that they have no competing interests.

## Authors’ contributions

 The study design was developed by JMZ, AS, MCP, CM, and MM. Data collection and coding were done by AS, MPC, CM, and MM. All authors performed the analysis, revised and approved the manuscript.

## Disclaimer

 The views expressed in the submitted article are those of the authors and not their funders.

## Funding

 Swedish Foundation for International Cooperation in Research and Higher Education (STINT), the South African National Research Foundation (NRF), and a network grant (ResearchLink) from the Swedish Research Council (Vetenskapsrådet, VR).

## Authors’ affiliations

^1^Department of Health Promotion and Education, School of Public Health, The University of Zambia, Lusaka, Zambia. ^2^Department of Health Policy and Management, School of Public Health, The University of Zambia, Lusaka, Zambia. ^3^The Ministry of Health, Lusaka, Zambia. ^4^Department of Epidemiology and Biostatistics, School of Public Health, The University of Zambia, Lusaka, Zambia. ^5^School of Public Health, University of the Western Cape, Cape Town, South Africa. ^6^South African Medical Research Council Health Services to Systems Unit, University of the Western Cape, Cape Town, South Africa.
